# Improving alignment accuracy on homopolymer regions for semiconductor-based sequencing technologies

**DOI:** 10.1186/s12864-016-2894-9

**Published:** 2016-08-22

**Authors:** Weixing Feng, Sen Zhao, Dingkai Xue, Fengfei Song, Ziwei Li, Duojiao Chen, Bo He, Yangyang Hao, Yadong Wang, Yunlong Liu

**Affiliations:** 1 Automation College, Harbin Engineering University, Harbin, Heilongjiang 150001, People’s Republic of China; 2 Center for Computational Biology and Bioinformatics, Indiana University School of Medicine, Indianapolis, IN 46202 USA; 3 School of Computer Science and Technology, Harbin Institute of Technology, Harbin, Heilongjiang 150001, People’s Republic of China

**Keywords:** Homopolymer, Ion Torrent/Proton, Bayesian, Alignment

## Abstract

**Background:**

Ion Torrent and Ion Proton are semiconductor-based sequencing technologies that feature rapid sequencing speed and low upfront and operating costs, thanks to the avoidance of modified nucleotides and optical measurements. Despite of these advantages, however, Ion semiconductor sequencing technologies suffer much reduced sequencing accuracy at the genomic loci with homopolymer repeats of the same nucleotide. Such limitation significantly reduces its efficiency for the biological applications aiming at accurately identifying various genetic variants.

**Results:**

In this study, we propose a *Bayesian* inference-based method that takes the advantage of the signal distributions of the electrical voltages that are measured for all the homopolymers of a fixed length. By cross-referencing the length of homopolymers in the reference genome and the voltage signal distribution derived from the experiment, the proposed integrated model significantly improves the alignment accuracy around the homopolymer regions.

**Conclusions:**

Besides improving alignment accuracy on homopolymer regions for semiconductor-based sequencing technologies with the proposed model, similar strategies can also be used on other high-throughput sequencing technologies that share similar limitations.

**Electronic supplementary material:**

The online version of this article (doi:10.1186/s12864-016-2894-9) contains supplementary material, which is available to authorized users.

## Background

The rapid development of high-throughput sequencing technologies leads to appearances of many innovative sequencing platforms [1, 2]. Ion Torrent and Ion Proton are semiconductor-based sequencing platforms that are primarily designed for personal genome sequencing [3, 4]. Different from sequencing techniques enriched with substitution errors [5, 6], Ion semiconductor sequencing platforms suffer from the inaccuracy in detecting the length of homopolymers repeats of the same nucleotide [7, 8]. These homopolymer errors often lead to the inaccurate local alignment results, and become a critical barrier against accurate detection of genomic variations [9–11] (http://www.broadinstitute.org/gatk/media/docs/Samtools.pdf).

The sequencing chemistry for the Ion semiconductor-based technology is that the incorporation of a deoxyribonucleotide (dNTP) into a strand of DNA couples with the release of a hydrogen ion, which changes the pH of the solution and then leads to the electronic voltage pulse in the ion sensor. Multiple identical bases on the DNA strand often result in the detection of multiple times of the baseline voltage corresponding to the measurements at mononucleotide loci [12]. The difficulty on the homopolymer length identification mainly results from the inaccurate measurement on the magnitude of the voltage pulse, which follows a signal distribution that can be dependent on multiple factors including the type of nucleotide, the length of homopolymer, and the relative position in the DNA template.

Thus far, several algorithms have been proposed in correcting the inaccurate homopolymer length identification, based on the raw data from the detected voltage signals for Ion semiconductor sequencing technologies. Lysholm designed a flow-space FAAST tool, where flowpeak information retrieved from detected voltage signals is utilized to improve accuracy of Smith-Waterman-Gotoh local alignment through correction of likely sequencing errors and thus obtain optimized homopolymer length [8]. However, since dedicatedly designed for the naïve Smith-Waterman-Gotoh algorithm, the method undertakes a heavy computing burden and limits the further application with other alignment programs. In addition, the parameter selection in the algorithm is ad hoc, and was not designed for maximizing the performance. Zeng designed a PyroHMMsnp algorithm, where a hidden Markov model (HMM) is built to recognize overcall or undercall status of homopolymers in a realignment process, and is used to deduce the most possible homopolymer lengths [7]. Similar with other refined alignment algorithms, this approach uses an EM-based strategy, which assumes the variant pattern on most of the reads at one specific loci follows the same distribution; this assumption maybe invalid for certain biological applications, such as the variant identification in cancer somatic tissues. In addition, PyroHMMsnp design does not have hidden state for mismatches, and therefore tends to mistakenly convert mismatches into INDELs. In this project, we aim to develop a simple computational strategy for improving the alignment accuracy by using the voltage signals, and relying only on the measurements of individual sequencing read.

In addition to the measured electrical voltage signals, it is evident that the reference genome contains significant amount of prior information that are not adequately considered by other methods. This is under the assumption that only a small percentage of nucleotides are different between two individuals; for human, it is about 1 % of whole genome nucleotides [13, 14]. Based on such consideration, we proposed a Bayesian-based integrated model to merge these two information sources to improve performance of homopolyer length identification. We demonstrate that our algorithm significantly outperformed Torrent Suite, the software package coupled with Ion Torrent and Proton Sequencers for accurately identifying the length of the homopolymer repeats, and therefore improved sequence alignment accuracy.

## Methods

### Ion Torrent sequencing

Different from imaging-based sequencing platforms, Ion semiconductor technology detects nucleotide composition using electronic sensors. During the sequencing process, the sensor detects released hydrogen ions when nucleotide incorporation occurs. The sensor then detects the pH change caused by hydrogen release, and translates such chemical signal to electrical voltage signal, which is proportional to the number of captured ions. Since one type of nucleotides is sequenced in one machine cycle, if homopolymer exists, the detected voltage level should reflect the length of homopolymer. Despite this simple principle, practically, however, the detected electrical voltage follows a distribution, and in many cases, may not accurately recapitulate the length of homopolymer. In order to design a bioinformatics strategy for correcting the length of homopolymers, we first systematically evaluate the signal distribution of the detected electrical voltage for all the nucleotide positions that share the same homopolymer length, same homopolymer nucleotide type (A, C, G, or T), and similar positions in the sequence reads. The original voltage signals for different nucleotides were extracted from the SFF file, which is exported from the Torrent Suite package.

### Bayesian inference of homopolymer length

We design a Bayesian-based model to infer the length of homopolymer based on the local genomic sequence context, including the homopolymer nucleotide type (N_i_ = A, C, G, or T), detected electrical voltage (V), and the nucleotide position in the sequencing reads (P_j_). In the current model, nucleotide position were classified into several categories.

1 $$ \begin{array}{l}P\left(L\Big|{N}_i,{P}_j,V\right)=P\left(V\Big|{N}_i,{P}_j,L\right)\ast P(L)/P(V)\\ {}=P\left(V\Big|{N}_i,{P}_j,L\right)\ast P(L)/{\displaystyle \sum_{i,j}\left(P\left(V\Big|{N}_i,{P}_j,L\right)*P\left({N}_i,{P}_j,L\right)\right)}\end{array} $$

In the equation, *P*(*V*|*N*_*i*_,*P*_*j*_*,L*) is the prior possibility of occurrence of a specific voltage *V* if given homopolymer length *L* under situation of nucleotide *N*_*i*_ and read position *P*_*j*_, while *P*(*L*) and *P*(*V*) respectively represent the probability of a specific homopolymer length *L*, and the probability of a specific voltage *V.* Both these two probabilities can be statistically derived from the entire sequencing data. In summary, *P*(*L*|*N*_*i*_*,P*_*j*_*,V*) is the probability of occurrence of a specific homopolymer length *L* if given sequencing voltage *V* under sequencing context of nucleotide *N*_*i*_ and read position *P*_*j*_.

### Integrated model to identify homopolymer length

The performance of statistical-based inference model highly relies on fully understanding the sources of detection error, and their intervened relationships. Additional biological information can be used to increase the detection accuracy. For most of the biological applications, it is reasonable to assume that only a small percentage of the nucleotide positions represent true variants comparing to the reference genome. Therefore, combining the homopolymer length in the reference genome with the statistically-inferred homopolymer length can potentially improve the detection accuracy. We therefore construct an integrated model by defining a score *S* for the homopolymer length at a specific homopolymer loci:

2 $$ S=W* \log \left(P\left(L\Big|{N}_i,{P}_j,V\right)\right)+\left(1-W\right)\ast Pen\left(L\Big|Seq\_ref\right) $$

In the model, *Pen*(*L*|*Seq_ref*) is a penalty value when mismatch occurs between the reference genome sequence and the deduced Ion Torrent sequence for a given homopolymer length *L*. The penalty value is defined as 0 for perfect match, −1 for substitution, and −2 for insertions/deletions. In order to ensure that the two types of measurements staying in a similar scale, Bayesian posterior probability *P*(*L*|*N*_*i*_*,P*_*j*_*,V*) is converted into logarithmic form. In Eq. 2, *W* is weighting factor to balance the contribution of the Bayesian model-derived score, and reference genome-derived penalty. For one homopolymer, its length *L* can be determined as the candidate with the largest score S_i_:

3 $$ L=\underset{i}{ \arg \max }{S}_i,i=2,3,4,5,6,\dots $$

For a specific assay, the weighting factor w will be determined by minimizing the identification error for the homopolymers whose length is known, such as samples also detected using other technologies.

## Results and discussion

### Data preparing

**Fig. 1:**
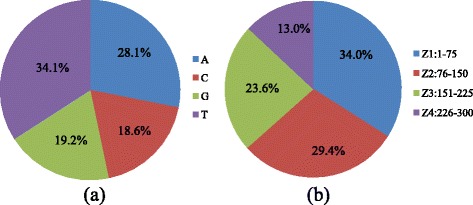
Profile of retrieved homopolymers. Profile of retrieved homopolymers according to (**a**) nucleotide type and (**b**) position in the sequencing reads

We have tested our model on one HapMap human dataset, NA11881, of which both Ion Torrent data and Illumina sequencing data is available. The availability of such dataset enables training and testing a statistical model for refining the identification of homopolymer length. The Ion Torrent dataset was generated in the Center for Medical Genomics at Indiana University, of which a targeted genomic region of 59 genes were sequenced. The overall targeted genomic area covers 90,918 basepairs. To derive the length of the homopolymer repeats, the electrical voltage signal for each influx nucleotide machine cycle was retrieved from the SFF file, where the type of the nucleotide (A, C, G, or T) is determined. Among 452,161 Ion Torrent sequencing reads that passed quality control, our assay detected 1,430,986 homopolymers with >1.5 voltage units; these regions are defined as homopolymer candidates that are used in further analysis. In order to further characterize the homopolymer profiles being identified in our dataset, we further examine the nucleotide composition of all the detected homopolymers, and their relative loci in the sequenced reads (Fig. 1). We observed enrichment of A and T homopolymers in our dataset, and evenly distributed homopolymer locations (except for the last location due to the varying lengths of the Ion Torrent reads).

Illumina sequencing data from the same individual, NA11881, is downloaded from the 1000 Genomes database (http://www.1000genomes.org/data). Due to the chemistry differences, Illumina technology is more accurate in detecting homopolymer lengths. We therefore use the dataset from the Illumina platform as gold standard when refining the length of homopolymer repeats.

### Distribution of detected voltage signals in homopolymer repeat regions

**Fig. 2:**
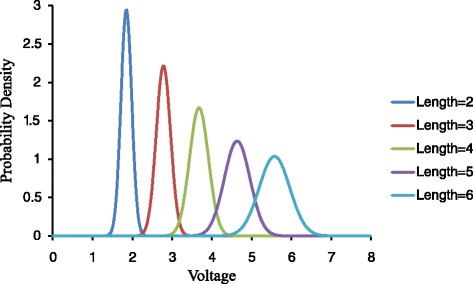
Prior possibilities of the detected voltages. Prior possibilities of the detected voltages when nucleotide type is A and position in the sequencing reads belongs to Z1

We examine the three factors that affect the distribution of the voltage signals on the homopolymer regions, the length of the homopolymer repeats, the types of homopolymer nucleotides, and the relative positions of the homopolymer repeats within a read. The homopolymer positions were classified into four zones depending on their distance from the beginning of the reads, Z1: 1–75 bp, Z2: 76–150 bp, Z3: 151–225 bp, and Z4: 226–300 bp. For the homopolymers with A nucleotides and appear in the first 75 bases, the retrieved signal distributions for each homopolymer length was demonstrated in Fig. 2. The ground truth for the homopolymer is derived from the Illumina dataset, which do not have apparent homopolymer issues. In Fig. 2, the horizontal axis is of voltage level and the vertical axis is of probability density for all the homopolymers of a fixed size. From left to right, there are five curves which correspond to the homopolymers with 2, 3, 4, 5, and 6 nucleotides. Here, the probability density of the voltages are fitted as in Gaussian distributions, where the mean values are 1.85, 2.78, 3.68, 4.64 and 5.57 respectively. It is observed that the standard deviation increases with homopolymer length. It increases from 0.14 for 2-base homopolymers to 0.38 for 6-base ones. A similar trend has been reported elsewhere [7]. This shift clearly suggests that the voltage signals become less specific with the homopolymer length increases. It is critical to consider these factors in the model for accurately inferring the homopolymer length. This is especially important for the sequencing reads with longer homopolymers.

**Fig. 3:**
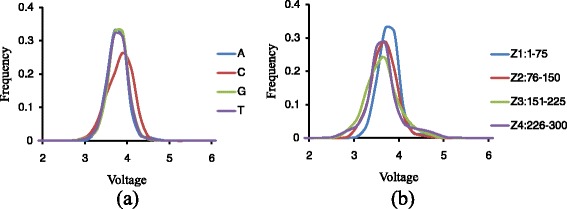
Other factors in Identification of homopolymer length. Other factors in identification of homopolymer length as (**a**) nucleotide type when homopolymer length is 4 and position in the sequencing reads belongs to Z1 and (**b**) position in the sequencing reads when homopolymer length is 4 and nucleotide type is A

Besides homopolymer length, we also observed differences in signal distribution for homopolymers with different nucleotide composition (A, C, G, or T) and their positions in the sequencing reads. As shown in Fig. 3a, when fixing the homopolymer length (*n* = 4) and homopolymer position zone (Z1, position 1–75 in the sequencing reads), we observed slightly different signal distribution for the homopolymers with different nucleotide compositions. Specifically, the C homopolymers tend to have higher signal values with mean signal intensity at 3.74, as comparing to other three nucleotides with average value at 3.68. In addition, the standard deviation for the C homopolymers (stdev = 0.30) are also slightly larger than the other three types (stdev = 0.24). Similar inconsistency was also observed for homopolymers that locate at different positions zones in the sequencing reads (Fig. 3b). Using all the AAAA as an example, the average signals tend to be higher in the beginning of the reads, and decrease toward the end of the reads. The average signal for Z1 to Z4 is 3.68, 3.57, 3.54, and 3.57 respectively. All these results suggest that the derived voltage signal is dependent on the homopolymer nucleotide composition and its relative positions in the sequencing reads, and should be considered while inferring the length of the homopolymers.

### Bayesian inference of homopolymer length

Motivated by these observations, we develop a Bayesian-based model in inferring the length of homopolymer based on the homopolymer length, their relative positions in the reads, and the detected voltage signal. Since the nucleotide composition includes A, C, G, T and the homopolymer positions are classified into four zones (Z1, Z2, Z3, Z4), in total, 16 Bayesian inference models are built based on the aforementioned prior signal distributions. In each model, the homopolymer length is identified if given a specific voltage level under a particular nucleotide type and position in sequencing read.

In fact, after calculation of prior signal distributions of different kinds of homopolymer lengths, the length of homopolymer can be simply decided using naïve counting from the measured electrical voltage or the k-nearest neighbors algorithm. That is to identify the length of one homopolymer according to its nearest distance to the mean values of different prior signal distributions. In such way, the number of identification errors is 169,212, or 11.82 % of the whole 1, 430,986 homopolymers.

Comparing to k-nearest neighbors algorithm, with our designed Bayesian inference models, the number of identification errors decrease to 71, 460, or 4.99 % of the whole homopolymers.

However, our Bayesian inference result cannot outperform that from the Torrent Suite, where the number of identification errors is 29,623, or 2.07 % of the whole homopolymers. This is due to the fact that significant training has been included the Torrent Suite algorithm, which is proprietary, and uses a large amount of genomic features.

### Identification of homopolymer length with Bayesian and reference genome information

Despite the superior performance of the Bayesian model comparing to naïve counting from the measured electrical voltage, both our model and output from the Torrent Suite, experience significant inconsistency based on our dataset with ground truth. Since genetic variants should only occur in a small percentage of genomic loci. We therefore hypothesize that using a combination of voltage signal with the guidance of the standard reference genome will significantly increase the detection accuracy.

**Fig. 4:**
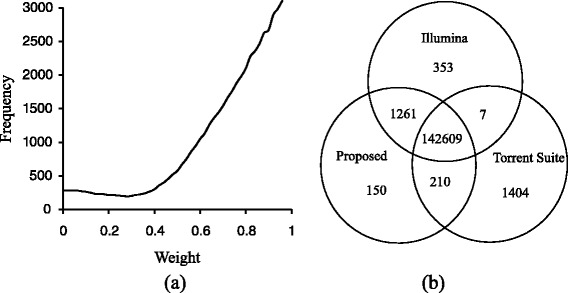
Identification result of homopolymer lengths. Identification result of homopolymer lengths when nucleotide type is A and position in the sequencing read belongs to Z1. The result is presented as (**a**) frequency of identification errors and (**b**) distribution of identification result

Using our proposed integrated model with Bayesian and reference genome information, we try to identify homopolymer length. In the integrated model, Eq 2, the weight parameter *W* was firstly optimized when the best identification result acquired (five cross validation) comparing to the results from the Illumina sequencing results. In Fig. 4a, the process of weight optimization is presented for the model under situation of nucleotide A and position Z1. When the weight is equal to 0, only reference genome information is referred in identification, while the weight equaling to 1 means only Bayesian inference information is used. Finally, the best weight value is equal to 0.28 when the least identification errors were found. The distribution of these errors is presented in Fig. 4b. Since the exact lengths of homopolymers were measured through Illumina platform, among 144,230 homopolymers under situation of nucleotide A and position Z1, lengths of 143,870 homopolymers were successfully identified by our proposed method with 360 errors. This is significant improvement comparing to using Bayesian model only. The performance also improved comparing to relying only on the reference genome, which enables to identify homopolymer-related variants from the sequencing data.

**Fig. 5:**
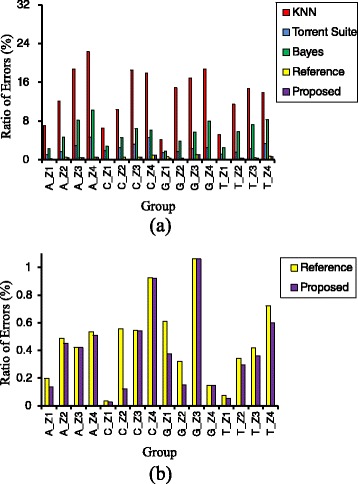
Comparison of identification results among different identification methods. Comparison of identification results among different identification methods according to (**a**) all methods and (**b**) two methods of only using reference information and the proposed method

All the optimized weights and corresponding identification errors are listed in Table 1. In Table 1, comparing with other methods, the best identification result is obtained with our proposed approach, which is also presented in Fig. 5.

**Table 1:** Identification errors of homopolymer length with different methods

No	Nt	Pos	Count	Errors (%)					
				KNN	Torrent suite	Bayesian	Reference	Proposed approach	
								Weight	Errors
1	A	1–75	144230	7.002	1.119	2.296	0.298	0.28	0.250
2	A	76–150	112776	12.121	1.651	4.722	0.489	0.34	0.453
3	A	151–225	97568	18.733	2.926	8.150	0.423	0.14	0.421
4	A	226–300	48033	22.292	4.655	10.259	0.535	0.24	0.510
5	C	1–75	88732	6.534	1.843	2.779	0.034	0.14	0.027
6	C	76–150	77650	10.382	2.489	4.595	0.556	0.36	0.121
7	C	151–225	63658	18.581	3.187	6.383	0.545	0.28	0.542
8	C	226–300	35736	17.910	4.600	6.159	0.926	0.30	0.923
9	G	1–75	97493	4.141	1.422	1.826	0.609	0.30	0.376
10	G	76–150	78192	14.874	1.623	3.864	0.322	0.32	0.152
11	G	151–225	64680	16.868	2.273	5.683	1.062	0.14	1.062
12	G	226–300	34116	18.754	2.492	7.985	0.147	0.12	0.147
13	T	1–75	156550	5.186	1.106	2.504	0.076	0.14	0.054
14	T	76–150	152034	11.446	1.571	5.780	0.342	0.30	0.297
15	T	151–225	111090	14.720	2.331	7.290	0.419	0.32	0.362
16	T	226–300	68448	13.912	3.315	8.240	0.723	0.28	0.599

“Count” means the number of each class of homopolymers. “KNN” means the method of K nearest neighbors. “Reference” means only reference information is used in the designed model(Weight *=* 0)

To show robustness of our proposed method, we also conducted analysis on one Ion Proton data(HapMap human dataset, NA12878) with the same pipeline and obtained the similar result (Additional file 1). Since more homopolymers retrieved in the Ion Proton data, their positions were classified into five zones depending on their distance from the beginning of the reads, Z1: 1–50 bp, Z2: 51–100 bp, Z3: 101–150 bp, Z4: 151–200 bp and Z5: 201–250 bp.

## Conclusions

As an important category of sequencing platform, Ion semiconductor-based technology has been widely utilized due to its good performance of faster and cheaper sequencing. However, the technology is far from perfect and suffers from the problem of homopolymer uncertain length. With Bayesian inference and reference genome information, an integrated model was designed to resolve such a problem. Bayesian inference of homopolymer length was first calculated from detected voltage signals. Merged with reference genome sequences information, the homopolymer length was eventually deduced. Compared to several known algorithms, the proposed method presents a greatly improved performance.

It should be noted that the proposed method is designed for refining the sequencing alignment based on individual sequencing read information. This is different from other approaches that rely on the coordinated information from all the reads that align to the same genomic region. Our strategy enables mapping the reads that contain variants in only a small percentage of DNA fragments, such as cancer genome. The general framework of our method can also be used for other sequencing technologies that contain significant amount of sequencing error around homopolymer regions, such as nanopore technology.

## Additional file

Additional file 1: Supplementary results. This file contains all supplementary results that are not covered in the manuscript, including 5 figures and 1 table on Ion Proton data. **Figure S1.** is about profile of retrieved homopolymers according to (a) nucleotide type and (b) position in the sequencing reads. **Figure S2.** is about prior possibilities of the detected voltages when nucleotide type is A and position in the sequencing reads belongs to Z1. **Figure S3.** is about other factors in identification of homopolymer length as (a) nucleotide type when homopolymer length is 4 and position in the sequencing reads belongs to Z1 and (b) position in the sequencing reads when homopolymer length is 4 and nucleotide type is A. **Figure S4.** is about identification result of homopolymer lengths when nucleotide type is A and position in the sequencing read belongs to Z1. The result is presented as (a) frequency of identification errors and (b) distribution of identification result. **Figure S5.** is about comparison of identification results among different identification methods according to (a) all methods and (b) two methods of only using reference information and the proposed method. **Table S1.** is about identification errors of homopolymer length with different methods. (PDF 396 kb)
