# Supplementary material for: Improving alignment accuracy on homopolymer regions for semiconductor-based sequencing technologies
Source: BMC Genomics. 2016 Aug 22;17(Suppl 7):521. doi: 10.1186/s12864-016-2894-9 (PMC5001236; doi:10.1186/s12864-016-2894-9)
Supplement: Additional file 1: — Supplementary results. This file contains all supplementary results that are not covered in the manuscript, including 5 figures and 1 table on Ion Proton data. Figure S1. is about profile of retrieved homopolymers according to (a) nucleotide type and (b) position in the sequencing reads. Figure S2. is about prior possibilities of the detected voltages when nucleotide type is A and position in the sequencing reads belongs to Z1. Figure S3. is about other factors in identification of homopolymer length as (a) nucleotide type when homopolymer length is 4 and position in the sequencing reads belongs to Z1 and (b) position in the sequencing reads when homopolymer length is 4 and nucleotide type is A. Figure S4. is about identification result of homopolymer lengths when nucleotide type is A and position in the sequencing read belongs to Z1. The result is presented as (a) frequency of identification errors and (b) distribution of identification result. Figure S5. is about comparison of identification results among different identification methods according to (a) all methods and (b) two methods of only using reference information and the proposed method. Table S1. is about identification errors of homopolymer length with different methods. (PDF 396 kb) [file 12864_2016_2894_MOESM1_ESM.pdf]

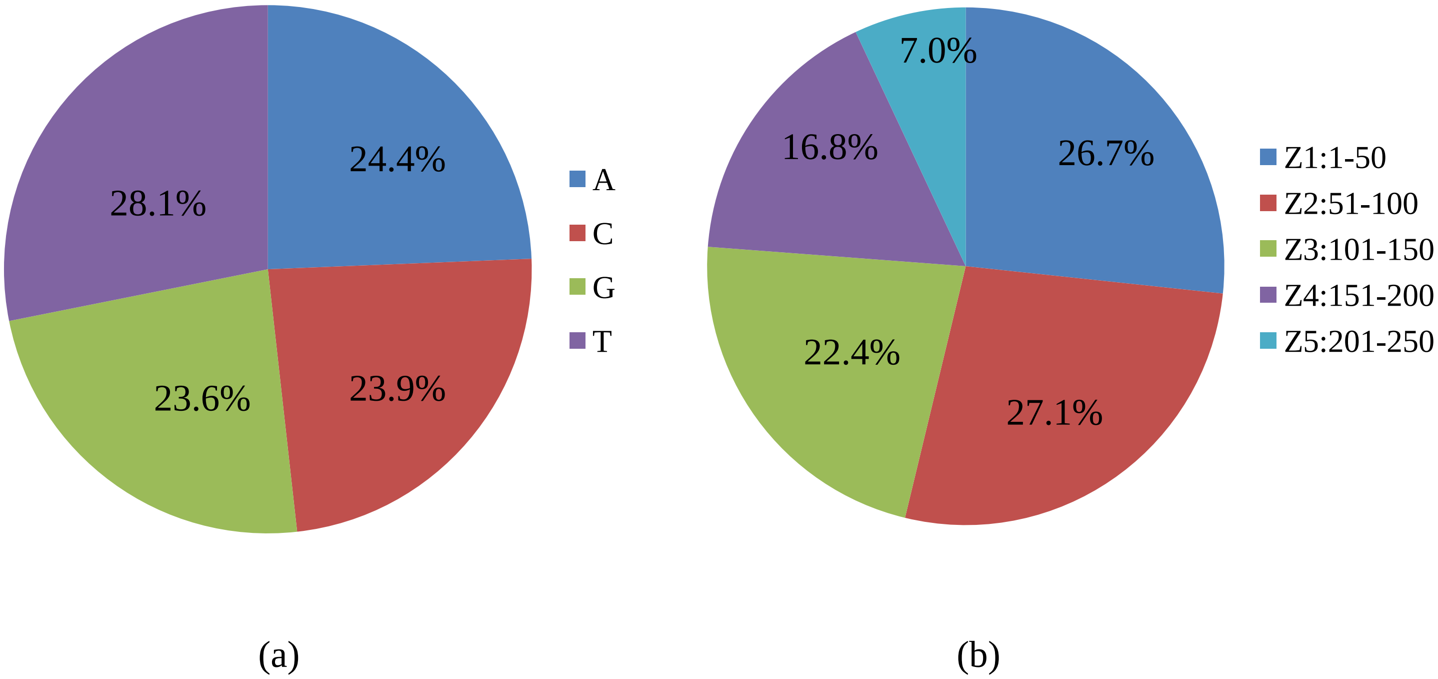
24.4%
23.9%
23.6%
28.1%
A
C
G
T
7.0%
26.7%
16.8%
22.4%
27.1%
Z1:1-50
Z2:51-100
Z3:101-150
Z4:151-200
Z5:201-250
(a)
(b)

Figure S1

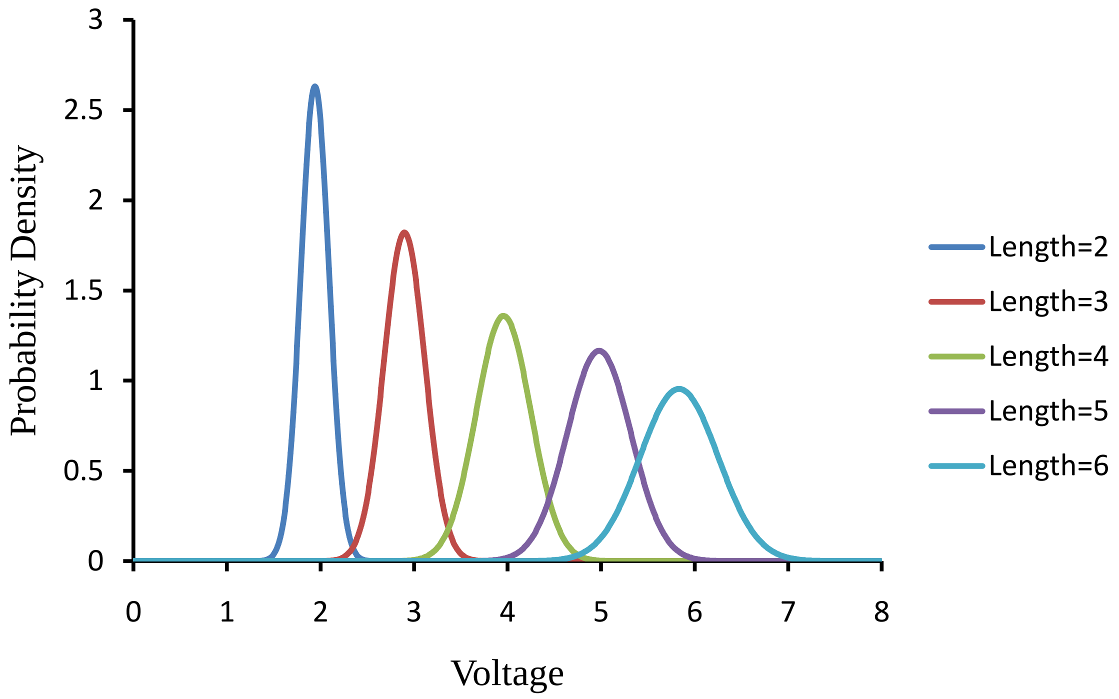

Probability Density
3
2.5
2
1.5
1
0.5
0
0
1
2
3
4
5
6
7
8
Voltage
Length=2
Length=3
Length=4
Length=5
Length=6

Figure S2

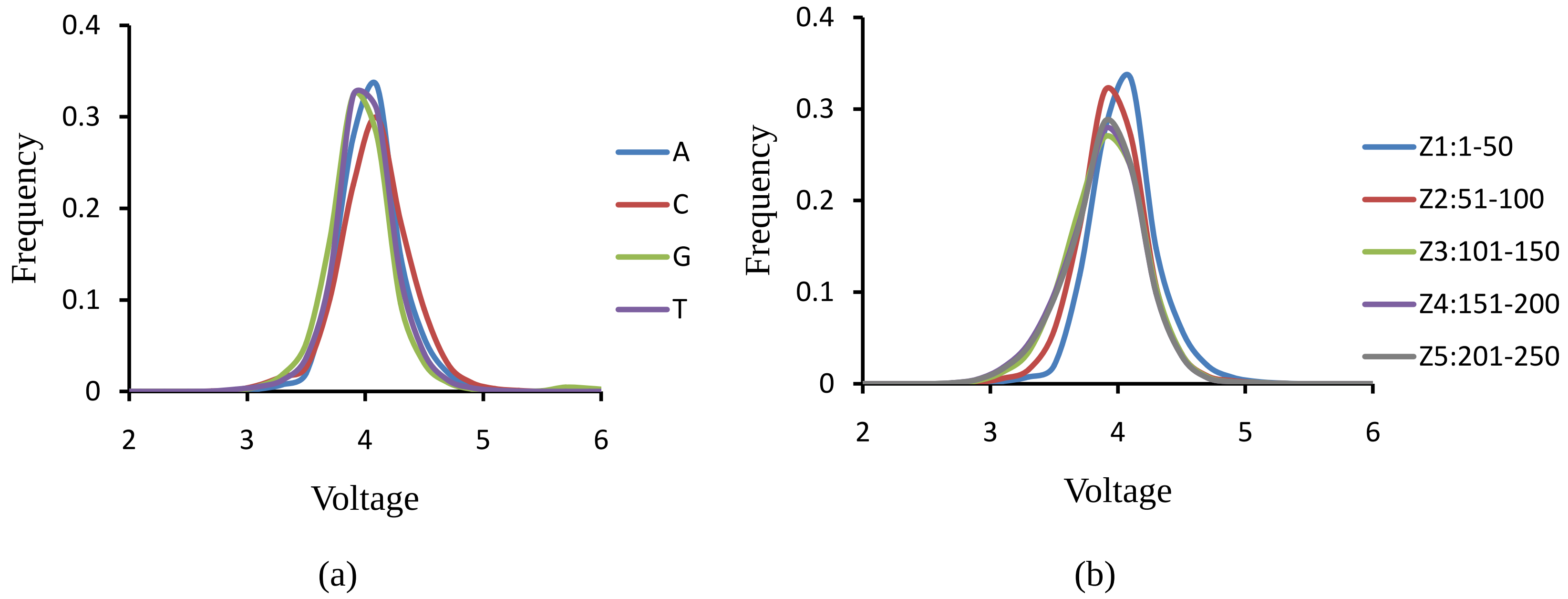
0.4
0.3
0.2
0.1
0
Frequency
2
3
4
5
6
Voltage
A
C
G
T
(a)
0.4
0.3
0.2
0.1
0
Frequency
2
3
4
5
6
Voltage
Z1:1-50
Z2:51-100
Z3:101-150
Z4:151-200
Z5:201-250
(b)

Figure S3

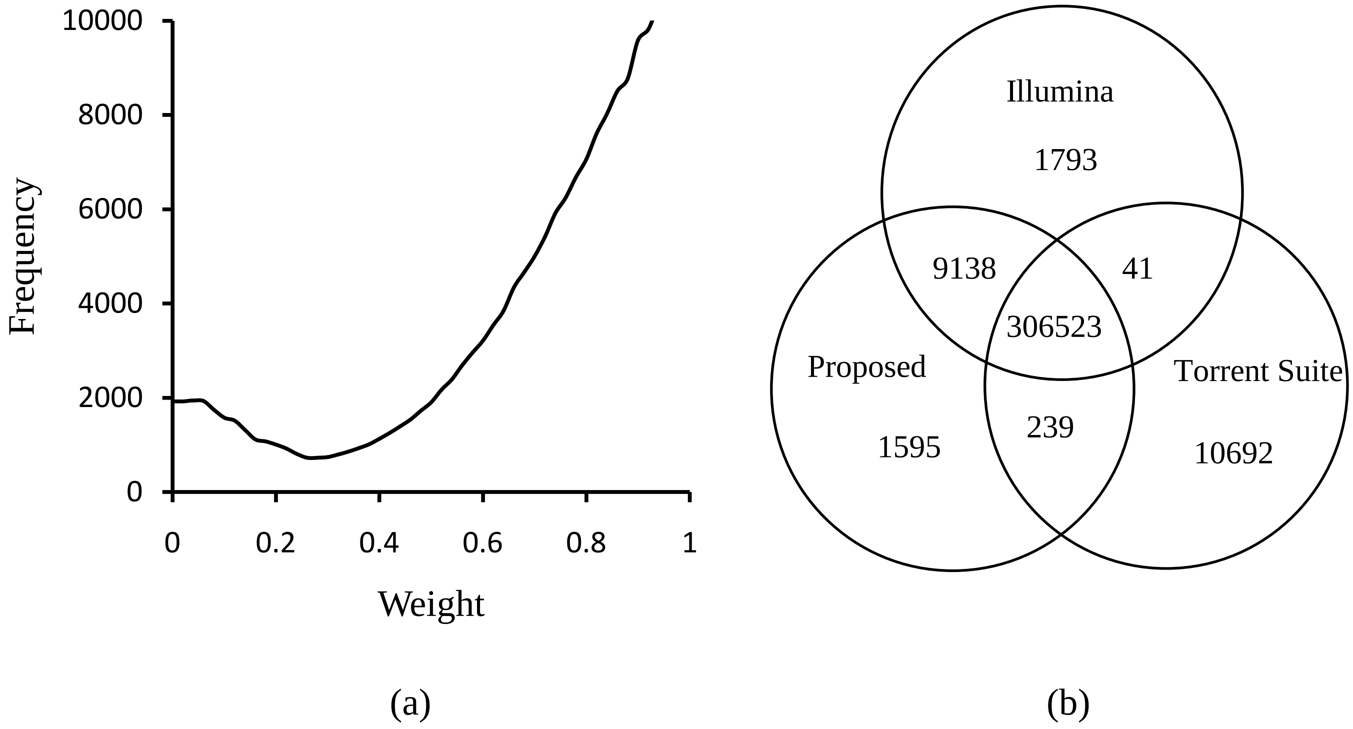

10000
8000
6000
4000
2000
0
0
0.2
0.4
0.6
0.8
1
Frequency
Weight
(a)
Illumina
1793
9138
41
306523
Proposed
Torrent Suite
239
1595
10692
(b)

Figure S4

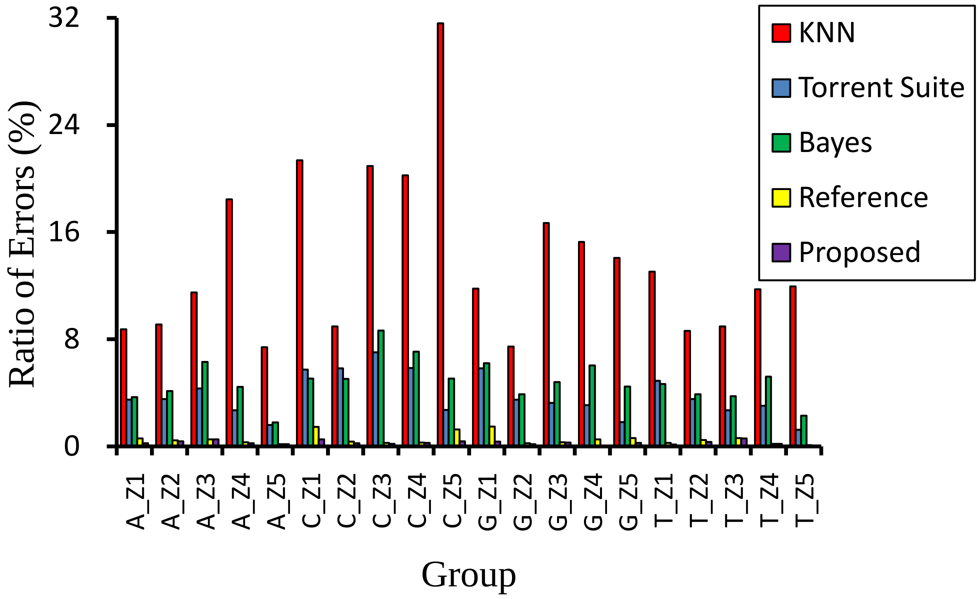
32
24
16
8
0
Ratio of Errors (%)
KNN
Torrent Suite
Bayes
Reference
Proposed
A_Z1
A_Z2
A_Z3
A_Z4
A_Z5
C_Z1
C_Z2
C_Z3
C_Z4
C_Z5
G_Z1
G_Z2
G_Z3
G_Z4
G_Z5
T_Z1
T_Z2
T_Z3
T_Z4
T_Z5
Group

(a)

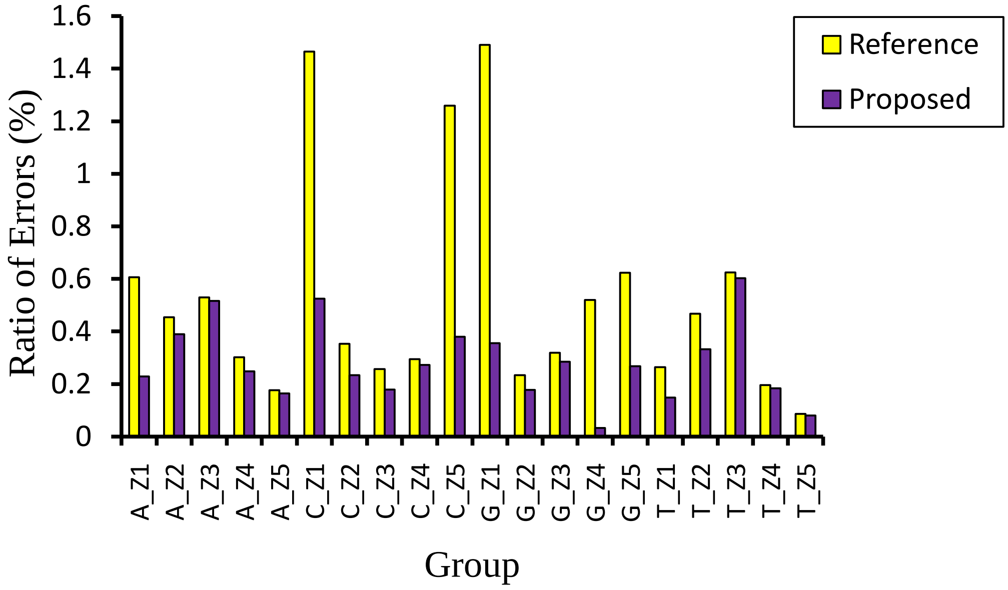
1.6
1.4
1.2
1
0.8
0.6
0.4
0.2
0
Ratio of Errors (%)
Reference
Proposed
A_Z1
A_Z2
A_Z3
A_Z4
A_Z5
C_Z1
C_Z2
C_Z3
C_Z4
C_Z5
G_Z1
G_Z2
G_Z3
G_Z4
G_Z5
T_Z1
T_Z2
T_Z3
T_Z4
T_Z5
Group

(b)

Figure S5

Table S1

No	Nucleotide	Position	Count	Errors(%)					
				KNN	Torrent Suite	Bayesian	Reference	Proposed approach	
								Weight	Errors
1	A	1-50	317495	8.755	3.494	3.681	0.606	0.28	0.229
2	A	51-100	282782	9.099	3.546	4.121	0.454	0.28	0.390
3	A	101-150	223362	11.484	4.332	6.315	0.531	0.18	0.516
4	A	151-200	151061	18.440	2.711	4.441	0.303	0.30	0.249
5	A	201-250	57559	7.403	1.600	1.793	0.177	0.28	0.165
6	C	1-50	265684	21.349	5.735	5.073	1.465	0.32	0.525
7	C	51-100	261905	8.956	5.839	5.035	0.354	0.30	0.234
8	C	101-150	223096	20.922	7.021	8.638	0.257	0.28	0.179
9	C	151-200	183784	20.242	5.854	7.074	0.295	0.20	0.273
10	C	201-250	76544	31.590	2.721	5.070	1.259	0.44	0.380
11	G	1-50	224681	11.779	5.832	6.219	1.490	0.22	0.356
12	G	51-100	289379	7.444	3.476	3.890	0.234	0.26	0.178
13	G	101-150	226858	16.667	3.239	4.811	0.320	0.30	0.285
14	G	151-200	181238	15.260	3.080	6.047	0.520	0.28	0.033
15	G	201-250	79010	14.079	1.805	4.455	0.624	0.34	0.268
16	T	1-50	322591	13.047	4.907	4.664	0.264	0.26	0.149
17	T	51-100	313631	8.631	3.527	3.886	0.468	0.32	0.332
18	T	101-150	276658	8.951	2.695	3.748	0.625	0.28	0.603
19	T	151-200	195991	11.727	3.029	5.219	0.196	0.16	0.184
20	T	201-250	83250	11.933	1.253	2.281	0.086	0.16	0.080
